# Monthly Reports

**Published:** 1803-04-01

**Authors:** J. Ricards

**Affiliations:** 68. Hatton Garden


### MONTHLY REPORTS.

*Cases admitted under the Care of the Surgeon of the Finsbury Dispensary, St. John's Square, Clerkenwell, from February 10, to March 10, 1803.*

| | | | |
|---|---|---|---|
| Phlegmone Tibiæ - - - | 1 | Lues - - - - - - - | 3 |
| ——— Sacri - - - | 1 | Gonorrhœa - - - - - | 1 |
| Erysipelas - - - - - - | 1 | Scrophula - - - - - - | 2 |
| Mastodynia - - - - | 2 | Steatoma - - - - - - | 1 |
| Abscessus - - - - - - | 1 | Sarcocele - - - - - - | 2 |
| Ulcera Artuum - - - | 8 | Incontinentia Urinæ - | 1 |
| Leucoma - - - - - - | 1 | Tinea - - - - - - - | 1 |
| Vulnera - - - - - - | 3 | Herpes - - - - - - - | 1 |
| Fractura Femoris - - | 1 | Hæmorrhois - - - - - | 1 |
| ——— Claviculæ - - | 1 | Bronchocele - - - - - | 1 |
| ——— Costæ - - - | 1 | Exostosis - - - - - - | 1 |
| Spasma Malleoli - - - | 1 | Clavus - - - - - - - | 1 |
| ——— Carpi - - - - | 1 | Eruptiones - - - - - - | 2 |
| Contusiones - - - - | 5 | Vacciola - - - - - - | 19 |
| Combustura - - - - | 1 | | — |
| Hydarthus Genu - - - | 2 | Total | 68 |

In the last Report was inserted the publication of the Medical Officers of this Dispensary, respecting the Vaccine Inoculation, and the order of the General Meeting for distributing its blessings among the lower ranks of the community, upon an enlarged scale; not to be confined within the limits of our district, but to embrace the extent of the metropolis, or as much wider a sphere as persons choose to come from to be inoculated; not circumscribed by a necessity of procuring a recommendation from a Governor, as in other cases of disease, but open to all persons by simple application.

The Inoculation commenced on the 28th of February; of course, only two inoculation days had occurred at the time of the closing of this Report. In that time nineteen children have been inoculated, and have passed through the disease in the usual favourable manner. Some time is required in making such an Institution generally known; but from the continually increasing number of applications, there is little doubt but that this Dispensary, being situated in a very populous neighbourhood, will be as fully attended, on this account, as any other place of gratuitous Inoculation in London or its vicinity.

It gives me great pleasure to perceive, that the prejudices of the poorer classes of the people are gradually declining; they embrace with avidity the good which is presented to them. As a proof of that, many of them are no longer sceptics, with respect to the salutary influence of the Vaccine disease, I have lately by several been informed of various unfortunate circumstances having attended or followed the Inoculation: but they have added, that they had no doubt but that the facts had been misrepresented to them; that these persons had not had the proper Vaccine disease; or that the unfavourable circumstances had been occasioned by the intervention of some other disease.

A considerable number of cases have recently occurred, of diseased joints, or what are commonly called white swellings; they are diseases which are very frequent among the poorer ranks of mankind. Although the true white swelling is undoubtedly of a scrophulous nature, yet diseased joints, unconnected with scrophula, or only secondarily connected with it, are constantly occurring, particularly as consequences of rheumatic affections: We see likewise very often, enlargements of the joints in old cases of syphilis, where much mercury has been given; I do not however think that the venereal action ever exerts itself upon the joints, but that the debilitated state of constitution occasioned by the united asthenic actions of a long continuance of the disease, and of large quantities of the specific remedy, are assistants to the variable state of our atmosphere in producing chronic rheumatism, and thickening of the ligaments.

The remedies for white swelling have been very various. The profession may, I think, consider themselves much indebted to Mr. Crowther for bringing into notice an application, by which the most beneficial process, that of a continued discharge, may be produced with much greater ease than by any other mode. In Dispensary practice, it is my constant custom, in these cases, to apply blisters to the joints, and suffer the discharge to continue for a considerable length of time, which is easily effected by the Savin cerate, the remedy above alluded to, when it is faithfully prepared. This mode of treatment, in by far the majority of cases, is productive of every good which can be wished.

*68. Hatton Garden.* J. RICARDS.

Mr. Ricards takes this opportunity of informing gentlemen of the profession, that, as much of the success of the Vaccine Inoculation depends upon the matter which is employed, he shall be happy to furnish them with recent virus, taken at the proper period, every Monday, (free of expence) if they will send their lancets or glasses to his house, on any of the preceding days, or on Monday mornings, before eleven o'clock, and send for them again in the evening. Gentlemen in the country, by sending them in letters (post paid) shall have them returned by the same conveyance.

